# Switching Characteristics and High-Temperature Dielectric Relaxation Behaviours of Pb(Zn_1/3_Nb_2/3_)_0.91_Ti_0.09_O_3_ Single Crystal

**DOI:** 10.3390/ma10040349

**Published:** 2017-03-28

**Authors:** Zhi Zhu, Xingui Tang, Yanping Jiang, Qiuxiang Liu, Tianfu Zhang, Wenhua Li

**Affiliations:** School of Physics & Optoelectric Engineering, Guangdong University of Technology, Guangzhou Higher Education Mega Centre, Guangzhou 510006, China; 15603013478@163.com (Z.Z.); ypjiang@gdut.edu.cn (Y.J.); liuqx@gdut.edu.cn (Q.L.); ztf143@163.com (T.Z.); liwenhuat@gdut.edu.cn (W.L.)

**Keywords:** PZNT single crystal, switching characteristics, oxygen vacancies, dielectric relaxation, pyroelectric coefficient

## Abstract

This work evaluated the resistance switching characteristics in the (100)-oriented Pb(Zn_1/3_Nb_2/3_)_0.91_Ti_0.09_O_3_ (PZNT) single crystal. The current hysteresis can be closely related to the ferroelectric polarization and we provided a possible explanation using a model about oxygen vacancies to analyze the mechanism of switching. The obvious frequency dispersion of the relative permittivity signified the relaxer-type behavior of the sample. The value of the relaxation parameter *γ* = 1.48 was estimated from the linear fit of the modified Curie-Weiss law, indicating the relaxer nature. High-temperature dielectric relaxation behaviors were revealed in the temperature region of 400–650 °C. In addition, under the measuring frequency of 10 kHz, *ε_r_* was tunable by changing the electric field and the largest tunability of *ε_r_* reached 14.78%. At room temperature, the high pyroelectric coefficient and detectivity figure of merit were reported.

## 1. Introduction

With flash memory approaching its scaling limit, the research and development of memory are urgent. Recently, various materials and novel devices have been studied for overcoming the scaling limit of conventional silicon-based memories [1]. New memory concepts include, for instance, magnetic random access memory (MRAM), ferroelectric random access memory and resistance switching random access memory (RRAM). A RRAM device is basically a two-terminal resistor device and it is attractive for its high speed, improved endurance, retention and excellent scalability for use in low-power nonvolatile memory applications. The resistance switching occurs between the low-resistance state and high-resistance state due to the electrically altered change in the resistance [2,3]. Some oxides and complex oxides, such as TiO_2−*x*_, ZnO_2_, NiO, TaO*_x_*, SrTiO_3_, Pr_0.7_Ca_0.3_MnO_3_, etc. [4,5,6,7,8,9,10,11,12,13,14,15], which have resistance switching characteristics, are under intense investigation due to their potential application in next-generation non-volatile memory [2,16]. In addition, the application of multiferroic materials has also gained great attention in resistance switching [17,18]. BiFeO_3_ is the best representative [19,20,21,22,23,24]. In the past two decades, unpoled relaxer ferroelectric (1 − *x*)Pb(Mg_1/3_Nb_2/3_)O_3_–*x*PbTiO_3_ and (1 − *x*)Pb(Zn_1/3_Nb_2/3_)O_3_–*x*PbTiO_3_ solid solutions have been extensively studied because of their excellent dielectric properties, piezoelectric properties and electromechanical properties compared with state-of-the-art lead zirconate titanate ceramics [25]. According to Bertram and Reck [26], at room temperature, (1 − *x*)Pb(Zn_1/3_Nb_2/3_)O_3_–*x*PbTiO_3_ exists in the rhombohedral (FE_r_) R3m phase when the value of *x* is less than 0.06. The rhombohedral R3m phase coexisted with the tetragonal (FE_t_) P4mm phase when the value of *x* was between 0.08 and 0.11. In addition, when *x* was greater than 0.11, (1 − *x*)Pb(Zn_1/3_Nb_2/3_)O_3_–*x*PbTiO_3_ presented a purely tetragonal P4mm phase. Otherwise, a metastable monoclinic phase (Pm) formed during the cooling process after crystal growth in the composition range of *x* between 0.06 and 0.11. Park and Shrout reported that (1 − *x*)Pb(Zn_1/3_Nb_2/3_)O_3_–*x*PbTiO_3_ single crystals were near the morphotropic phase boundary (MPB) when the value of *x* was about 0.09 [27]. They pointed out that (Pb(Zn_1/3_Nb_2/3_)_0.91_Ti_0.09_O_3_ (PZNT) single crystal exhibited two main phases: a rhombohedral- tetragonal ferroelectric (FE) phase between room temperature and 180 °C, and a cubic paraelectric (PE) phase above 180 °C. The PZNT single crystal had two major structures near the MPB: the rhombohedral phase (R3m) and the tetragonal phase (P4mm), and the temperature of the rhombohedral-tetragonal phase transition was about 90 °C. The phase transition could be induced by varying the temperature, electric field or hydrostatic pressure [25,28,29]. Bao et al. reported the dielectric relaxation in the PZNT single crystal at low temperatures, and the relaxation process could be attributed to the freezing of ferroelectric macrodomain walls induced by the pinning of point defects [30]. There is a great deal of literature on the resistance switching characteristics of thin-film metal oxides, but very little work related to ferroelectric single crystals. Pilch et al. reported the resistance switching in a defect-containing ferroelectric PbTiO_3_ crystal [31]. For these reasons, research on resistance switching characteristics of the PZNT single crystal is meaningful.

Although many works regarding the PZNT single crystal have already been published, the resistance switching characteristics and high-temperature dielectric relaxation phenomenon for the PZNT single crystal were beyond the interest of researchers up to now. In this work, the resistance switching characteristics and mechanism were discussed, and the high-temperature dielectric relaxation phenomenon, the dielectric tunability and the pyroelectric properties of the (100)-oriented PZNT single crystal were also reported.

## 2. Experimental Section

The PZNT single crystal was grown using a modified Bridgman method [27]. The sample oriented along the (100) direction was prepared with dimensions of 5 mm × 5 mm × 1 mm and electroded with silver.

The polarization-electric-field (*P*–*E*) hysteresis loops was obtained using a Sawyer-Tower circuit by applying a sinusoidal input signal with a frequency of 1 Hz. The reference capacitor used in the measurement is 5 μF. The *I*–*V* curve was measured by a Radiant Technologies Precision premier II Ferroelectric Tester (Albuquerque, NM, USA). The temperature dependence of relative permittivity was measured by a precision LRC meter (Agilent E4980A, Agilent Technologies Inc., Santa Clara, CA, USA) in the temperature range of 20–650 °C with the heating rate of 2 °C/min. The biased temperature dependence of dielectric response was measured using a blocking circuit, a dc power source (Keithley 6517A, Keithley Instruments Inc., Solon, OH, USA) and a multi–frequency LCR meter (Model SR720 of Stanford Research Systems, Stanford University, Stanford, CA, USA) at frequencies of 10 kHz and linear temperature change was adopted as 1 °C/min in the biased heating from the temperature range of 25–240 °C. The tunability was measured using a blocking circuit, a dc power source (Keithley 6517A) and a multi-frequency LCR meter (Model SR720 of Stanford Research System) at room temperature.

Furthermore, the temperature dependence of pyroelectric coefficient was measured by a dynamic technique [32,33]. At a certain temperature *T*_0_, the sample temperature was sinusoidally modulated *T*(*t*) = *T*_0_ + *T_~_*sin 2π*ft* ] with frequency *f* = 5 mHz and amplitude *T_~_* = 1 K using a Peltier element. The pyroelectric current signal was amplified with an electrometer and the 90° out of phase component of current with respect to the temperature modulation was measured with a lock-in amplifier. After setting to a new temperature *T*_0_ the sample was kept at *T*_0_ for 15 min for the signal to become stable before the pyroelectric measurement was performed. PZNT single crystal was first poled at room temperature by applying an electric field of amplitude 10 kV/cm for 5 min and then short circuited circuited at room temperature overnight.

## 3. Results and Discussion

**Figure 1:**
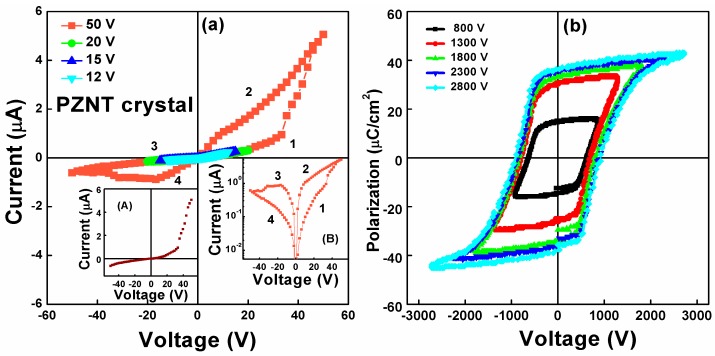
(**a**) The *I*–*V* curves for the PZNT single crystal by decreasing the sweep range step by step; Inset (A) shows the obvious diode-like rectifying *I*–*V* characteristic, indicating a diode behavior; Inset (B) shows the *I*–*V* curves plotted on semilogarithmic scales; (**b**) The *P*–*E* hysteresis loops for the PZNT single crystal with various voltages.

The current-voltage (*I*–*V*) curves for the (100)-oriented PZNT single crystal obtained by decreasing the sweep range step by step are shown in Figure 1a. In the range of ±50 V, the test voltage was swept at a constant rate from 0–50 V, then to −50 V, before returning to 0 V. The numbers in the figure denote the sequence of the voltage sweeps. An asymmetry in the current can be observed in the figure. In addition, we can find that the* I*–*V* curves show distinct hysteresis behavior, indicating the sample exhibited obvious resistance switching characteristics. Moreover, in the inset (A) of Figure 1a, the *I*–*V* segment shows an obvious diode-like rectifying *I*–*V* characteristic, indicating a diode behavior.

The *I*–*V* curves plotted on semilogarithmic scales are shown in the inset (B) of Figure 1a. When the sweeping positive bias increased from 0 V to about 33.3 V, the resistance switching was in the high-resistance state (HRS), with a low current. When the voltage was beyond 33.3 V, the current increased quickly and attained a maximum (around 5 µA) with the voltage reached 50 V, indicating that the resistance switching “turns on” and switches from the HRS to the low-resistance state (LRS). As the voltage swept back from 50 V to about −33.3 V, the resistance switching remained in the LRS. As the resistance switching continued its negative bias, the current stabilized at around 0.7 µA until the sweeping bias exceeded −33.3 V, which led to a transition from the LRS to HRS. Finally, when the sweeping voltage returned, going from −50 to 0 V, the resistance switching remained in the HRS [34,35]. In addition, the resistance switching ratio was 229 at 2.08 V.

The mechanism of resistance switching characteristics is not yet perfectly understood. Despite this, there were some phenomenological models to explain these characteristics, namely the Schottky barrier model [36,37], space charge [38,39], the electrically conducting filamentary model [40,41], and the Mott transition [42]. Jeon et al. [43] reported that resistance switching characteristics are caused by the change in the Schottky potential based on the results from the first-principles calculations. It is worth mentioning that the observed ferroelectric resistance switching behavior in our crystal would be different from that observed in some ferroelectric tunneling junctions [44,45], because the tunneling current can only be taken into account for the ultrathin ferroelectric materials, not for our crystal with a thickness of 1 mm.

In the range of ±12, ±15 and ±20 V, the *I*–*V* curve was almost symmetrical and without hysteresis, as shown in Figure 1a. Then, as the voltage increased to ±50 V, obvious hysteresis and resistance switching characteristics were observed. It was indicated that the current hysteresis and diode-like behavior can be triggered and switched at a high applied field. Figure 1b shows the *P–E* hysteresis loops for the PZNT single crystal with various voltages, indicating that the sample exhibits good ferroelectricity. It is worth mentioning that the *P–E* hysteresis loops starts at around ±45 V (not marked in the figure). According to the report of BiFeO_3_ [46], because the current and ferroelectric polarization exhibited the hysteresis phenomenon at a high applied field, the current hysteresis can be closely related to the ferroelectric polarization.

The polarization mechanism of ferroelectric materials includes displacement polarization and turning-direction polarization. In the process of the preparation of samples, lead vacancies will appear inevitably due to the volatility of lead, leading to the oxygen vacancies (OVs) due to charge neutrality restrictions. It is well known that the ionization of OVs will create conducting electrons in perovskite-structure oxides during the process of preparation at high temperatures, written as:

*Vo* → *Vo*• + *e'* (1)

*Vo*• → *Vo*•• + *e'* (2)

where *Vo•*, *Vo••* are single-ionized and double-ionized OVs, respectively. The current occurs by electron injection from one electrode affected by the concentration of OVs near the interface. So, according to the current study, we preferred to believe that the resistance switching characteristics of the PZNT single crystal could be caused by the change in the oxygen vacancies concentration at the metal/oxide interface by the electrically controlled electron injection.

In order to describe the processes underpinning resistance switching, the displacement and migration of OVs near the bottom electrode (BE) were introduced. Displacement was defined as the reversible movement of OVs under electrical bias (the OVs cannot get enough energy to go over the Schottky barrier), where upon removal of the applied voltage the OVs return to their initial locations. Migration occurred when the field-driven movement of the vacancies was not reversible upon removal of the bias (the OVs can get enough energy to go over the Schottky barrier) [47].

**Figure 2:**
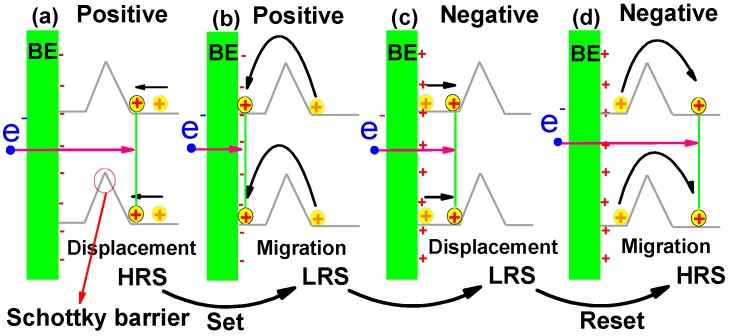
The displacement of OVs (**a**) and migration of OVs (**b**) under positive bias to enhance the electron injection; The displacement of OVs (**c**) and migration of OVs (**d**) under negative bias to reduce the electron injection.

A model, shown in Figure 2a–d, was set up to explain the resistance switching characteristics of the PZNT single crystal. The yellow circles with plus signs in the model represented OVs. When the sweeping positive bias increased from 0 V to 33.3 V, the OVs could not get enough energy to go over the Schottky barrier. The positive bias displaces OVs towards BE (displacement of OVs) to enhance the electron injection, as shown in Figure 2a. However, when the bias was beyond 33.3 V, the OVs surmounted the Schottky barrier to BE (migration of OVs) because they obtained enough energy, and it greatly increased the electron injection, as shown in Figure 2b. Consequently, the current increased quickly, the resistance switching “turned on” and the switches changed their state from the HRS to the LRS. The state would remain until the bias reversal. As the voltage swept from 0 V to around −33.3 V, the state still remained at the LRS, but the current appeared asymmetric due to the displacement of OVs, as shown in Figure 2c. When the negative bias kept increasing, the resistance switching returned to the HRS because of the migration of the OVs. Finally, the resistance switching returned to the original state when the bias returned to 0 V.

**Figure 3:**
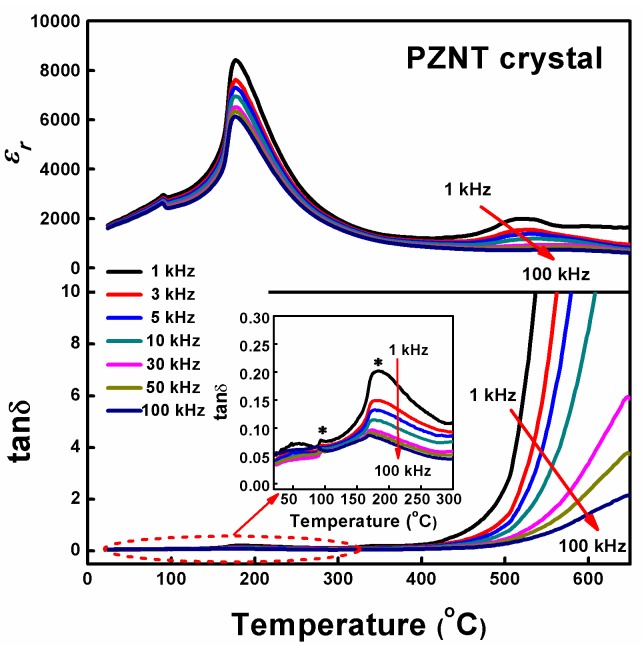
The relative permittivity *ε_r_* and dielectric loss tan*δ* as a function of temperature for the PZNT single crystal measured at different frequencies. The inset shows the dielectric loss tan*δ* in the temperature range between 20–300 °C

In the heating temperature range from 20–650 °C at a rate of 2 °C min^−1^, the temperature dependence of the relative permittivity *ε_r_* and the dielectric loss tan*δ* of the PZNT single crystal at various frequencies (from 1–100 kHz) are shown in Figure 3. The inset shows the dielectric loss tan*δ* in the temperature range between 20–300 °C. Permittivity curves for various frequencies exhibit organized (the relative permittivity *ε_r_* decreased with the increasing frequency) and the dielectric peaks are located at the temperature of about 177 °C (temperature of the maximum dielectric permittivity (*T_m_*)) and *T_m_* is unchanged with the increasing frequency, indicating a phase transition from the FE phase to the PE cubic phase. Seung-Eek Park et al. reported that the phase transition temperature was 180 °C [25]. In the temperature of *T_m_*, the values of the dielectric peak *ε_m_* could be as high as 8420 at the measurement frequency of 1 kHz. With increasing the frequency from 1–100 kHz, the value of *ε_m_* decreased from 8420 to 6135. Specially, there was another dielectric anomaly peak that took place at about 90 °C, indicating a phase transition from the FE_r_ phase to the FE_t_ phase. The same phenomenon had also been reported by Hosono et al. [48]. Obviously, we can observe (inset of Figure 3) the same phase transformation from the dielectric constant. However, it was worth noting that, at the high temperature range (400–650 °C), the permittivity curves presented a high-temperature relaxation phenomenon that looked like the behavior of a diffuse phase transition and the value of tan*δ* became large, because the space charge polarization or the conductivity of the insulating ceramics increased with the increase in temperature.

**Figure 4:**
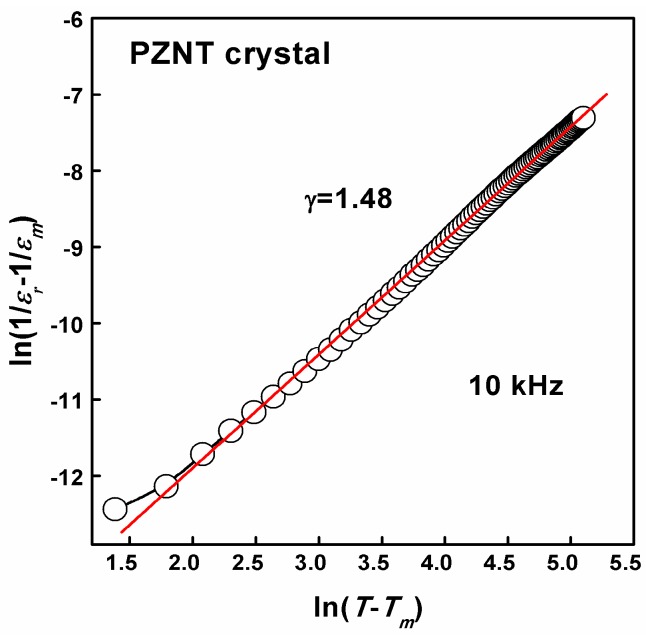
Plot of ln(1/*ε_r_ −* 1/*ε_m_*) as a function of ln(*T* − *T_m_*) for the PZNT single crystal measured at 10 kHz.

From Figure 3, the obvious frequency dispersion of the dielectric constant can be observed, and this phenomenon signifies the typical relaxer behavior of the present specimen. The dielectric characteristics of relaxor ferroelectric materials are well known to deviate from the typical Curie-Weiss behavior, and can be described by a modified Curie-Weiss relationship [49]:

(3) $$1/\varepsilon_{r}-1/\varepsilon_{m}={\left(T-T_{m}\right)}^{\gamma }/C_{1},1\leq \gamma \leq 2$$

where *γ* and *C*_1_ are assumed to be constant. For *γ* = 1, a normal Curie-Weiss law was obtained, and a complete diffuse phase transition was described for *γ* = 2 [50]. The plots of ln(1/*ε_r_* − 1/*ε_m_*) versus ln(*T − T_m_*) with a frequency of 10 kHz are shown in Figure 4. We can get the value of *γ* = 1.48 by fitting the experimental data. Values of *γ* in this work were found to vary from 1.31 to 1.62 in the frequency range from 1–300 kHz. This also supported the evidence of the relaxer nature of the PZNT single crystal.

**Figure 5:**
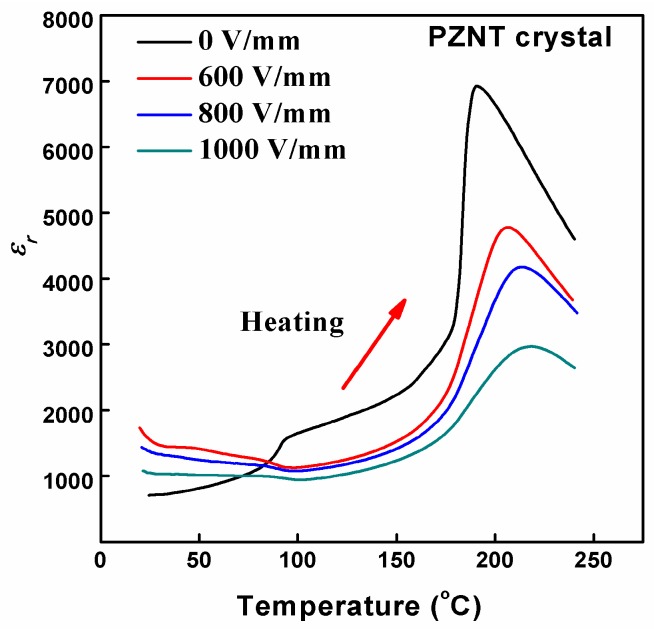
Temperature dependence of the relative permittivity (*ε_r_*) of the PZNT single crystal under various electric fields at 100 Hz.

At 100 Hz, the temperature dependence of *ε_r_* for the PZNT single crystal during the heating process under different electric fields is shown in Figure 5. The curves of the temperature dependence of *ε_r_* for the PZNT single crystal were lower and wider as the electric field increased, indicating that the single crystal went through a field-induced phase transition. When the value of the applied field was 0 kV/mm, the maximum relative permittivity (*ε_m_*) was 6934 at about 190 °C and the *ε_m_* decreased from 6934 to 2970 with the applied field increasing from 0 to 1 kV/mm. Particularly, the *T_m_* presented a different value under different applied electric fields, suggesting that the single crystal underwent a second-order phase transition. The values of *ε_m_* and *T_m_* are listed in Table 1. The result clearly indicates a broadening of the dielectric peak due to the diffuse ferroelectric-paraelectric phase transition.

**Figure 6:**
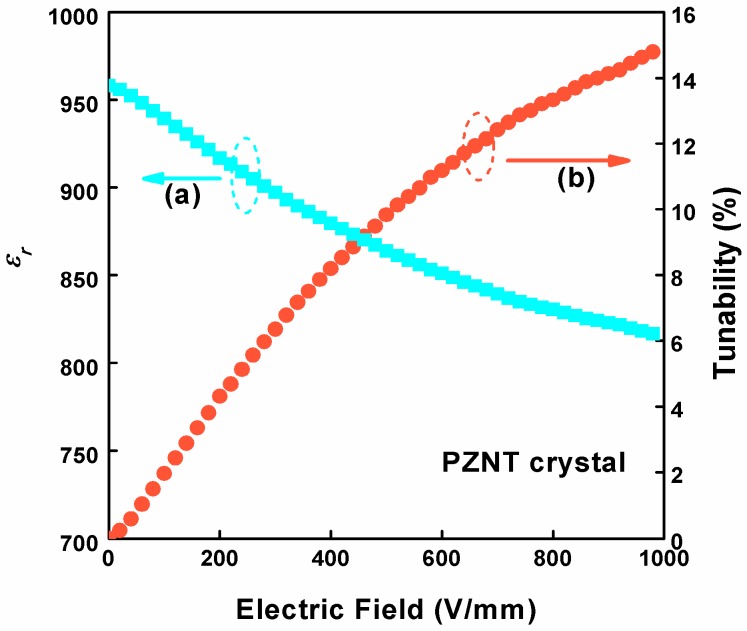
At room temperature, 10 kHz of AC bias and composition dependence of (**a**) the relative permittivity *ε_r_*; (**b**) the tunability of *ε_r_* of the PZNT single crystal.

Dielectric tunable materials have a wide range of applications, such as in phase shifters, oscillators, filters, etc. [51]. From the above discussion, *ε_r_* was found to be tunable by changing the electric field. The *ε_r_* and the tunability of *ε_r_* are shown in Figure 6, respectively. The test was conducted under different electric fields at 10 kHz at room temperature and the applied electric fields increased from 0 to 1000 V/mm. The tunability of *ε_r_* is defined as [*ε_r_*(0) − *ε_r_*(*E*)] × 100%/*ε_r_*(0), where *ε_r_*(0) and *ε_r_*(*E*) are the *ε_r_* values when the electric field is zero and *E*, respectively. The results revealed that the largest tunability of *ε_r_* was 14.78%. With the increase of the electric field, the *ε_r_* decreased gradually and the tunability increased, respectively [52,53].

**Figure 7:**
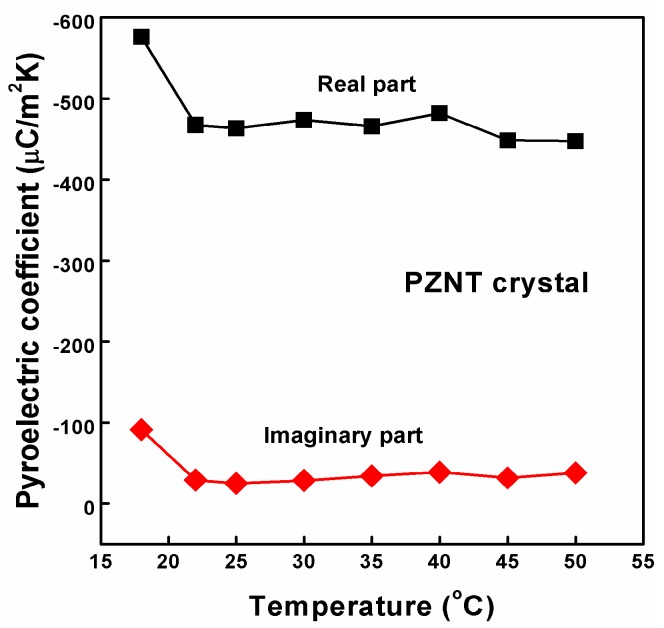
The pyroelectric coefficient as a function of temperatures for the PZNT single crystal.

The pyroelectric coefficients as a function of the temperature for the PZNT single crystal are shown in Figure 7. The real part of the pyroelectric coefficients increases slowly from −576.1 to −447.4 μC/m^2^K with increasing temperatures from 18–50 °C, respectively. The imaginary part of the pyroelectric coefficients are pyroelectric losses. At a room temperature of 25 °C, the value of the pyroelectric coefficient was −463.3 μC/m^2^K, and the absolute value of the pyroelectric coefficient (463.3 μC/m^2^K) was higher than that of the graded PZT films on Pt-coated silicon substrates (202–250 μC/m^2^K) and Ba_3_Nb_2_O_8_ ceramic (103 μC/m^2^K) [32,54]. A useful comparative figure of merit (*FOM*) used in comparing pyroelectric materials is defined as *F*_D_ = |*P*|/*c*(*ε_r_ε*_0_tan*δ*)^1/2^, where *c* is the heat capacity per unit volume (*c* = 2.5 J/cm^3^K) [55], *ε_r_* is the relative permittivity, *ε*_0_ is the permittivity of the vacuum and tan*δ* is the dissipation factor. The *F_D_* value is 8.77 × 10^−6^ Pa^−0.5^ at 1 kHz. This result implies that the PZNT single crystal can be a promising material for pyroelectric array sensor applications.

## 4. Conclusions

In conclusion, this work has shown the resistance switching characteristics in the (100)-oriented PZNT single crystal. The current hysteresis can be closely related to the ferroelectric polarization and we provided a possible explanation with a model of oxygen vacancies to analyze the mechanism of switching. In the process of heating, the temperature of the rhombohedral-tetragonal phase transition was about 90 °C and the temperature of the FE-PE phase transition was about 177 °C. The obvious frequency dispersion of the relative permittivity signified the relaxer-type behavior of the sample, and the value of the relaxation parameter *γ* = 1.48, estimated from the linear fit of the modified Curie-Weiss law, indicated the relaxer nature. High-temperature dielectric relaxation behaviors were revealed in the temperature region of 400–650 °C. In addition, under the measuring frequency of 10 kHz, we found that *ε_r_* was tunable by changing the electric field and the largest tunability of *ε_r_* reached 14.78%. At room temperature, the pyroelectric coefficient and the figure of merit *F_D_* were −463.3 μC/m^2^ K and 8.77 × 10^−6^ Pa^−0.5^, respectively.

## Figures and Tables

**Table 1:** The maximum relative permittivity and corresponding temperature under different electric fields.

Electric Field (V/mm)	0	600	800	1000
*ε_m_*	6934	4779	4179	2970
*T_m_* (°C)	190	206	214	218
